# Functional and Clinical Significance of Dysregulated microRNAs in Liver Cancer

**DOI:** 10.3390/cancers13215361

**Published:** 2021-10-26

**Authors:** Po-Shuan Huang, Chia-Jung Liao, Ya-Hui Huang, Chau-Ting Yeh, Cheng-Yi Chen, Hui-Chi Tang, Cheng-Chih Chang, Kwang-Huei Lin

**Affiliations:** 1 Department of Biochemistry, College of Medicine, Chang Gung University, Taoyuan 333, Taiwan; leo_6813@msn.com (P.-S.H.); L329735@ms49.hinet.net (C.-J.L.); 2 Department of Biomedical Sciences, College of Medicine, Chang-Gung University, Taoyuan 333, Taiwan; 3 Liver Research Center, Chang Gung Memorial Hospital, Taoyuan 333, Taiwan; e1249060@gmail.com (Y.-H.H.); chauting@cgmh.org.tw (C.-T.Y.); 4 Department of Cell Biology and Anatomy, College of Medicine, National Cheng Kung University, Tainan 701, Taiwan; cychen@gs.ncku.edu.tw; 5 Department of Biochemistry and Molecular Biology, McGovern Medical School, University of Texas Health Science Center at Houston, Houston, TX 77030, USA; Hui-Chi.Tang@uth.tmc.edu; 6 Department of General Surgery, Chang Gung Memorial Hospital at Chia-yi, Chia-yi 613, Taiwan; 7 Research Center for Chinese Herbal Medicine, College of Human Ecology, Chang Gung University of Science and Technology, Taoyuan 333, Taiwan

**Keywords:** liver cancer, microRNA, clinical

## Abstract

**Simple Summary:**

Liver cancer has a high mortality rate. Here, we retrospectively discuss the current progress and dilemmas in the clinical research and treatment of liver cancer. We primarily focus on microRNAs because of their extremely high value in applications and research. We discuss whether microRNAs can be used for the development of better biomarkers and/or therapeutic drugs, and address the difficulties, requirements for improved diagnostic technologies, and side effects related to microRNA-based drugs.

**Abstract:**

Liver cancer is the leading cause of cancer-related mortality in the world. This mainly reflects the lack of early diagnosis tools and effective treatment methods. MicroRNAs (miRNAs) are a class of non-transcribed RNAs, some of which play important regulatory roles in liver cancer. Here, we discuss microRNAs with key impacts on liver cancer, such as miR-122, miR-21, miR-214, and miR-199. These microRNAs participate in various physiological regulatory pathways of liver cancer cells, and their modulation can have non-negligible effects in the treatment of liver cancer. We discuss whether these microRNAs can be used for better clinical diagnosis and/or drug development. With the advent of novel technologies, fast, inexpensive, and non-invasive RNA-based biomarker research has become a new mainstream approach. However, the clinical application of microRNA-based markers has been limited by the high sequence similarity among them and the potential for off-target problems. Therefore, researchers particularly value microRNAs that are specific to or have special functions in liver cancer. These include miR-122, which is specifically expressed in the liver, and miR-34, which is necessary for the replication of the hepatitis C virus in liver cancer. Clinical treatment drugs have been developed based on miR-34 and miR-122 (MRX34 and Miravirsen, respectively), but their side effects have not yet been overcome. Future research is needed to address these weaknesses and establish a feasible microRNA-based treatment strategy for liver cancer.

## 1. Introduction

Hepatocellular carcinoma (HCC) is the leading cause of cancer-related mortality in Taiwan. One of the key challenges in therapy is the lack of an efficient assay system that can detect HCC at the early stages to allow timely treatment. Other than alpha-fetoprotein (AFP), no suitable diagnostic markers are available for HCC. However, other physiological factors often interfere with the detection of AFP, and its accuracy is insufficient to correctly reflect the risk of liver cancer. MicroRNAs are small non-coding RNAs that affect the stability and post-transcription of mRNAs through binding the 3′ untranslated regions (3′UTR) of target genes to regulate their expression, consequently influencing multiple cellular functions, including migration, proliferation, angiogenesis, and apoptosis. Relative to coding RNAs, microRNAs have high value in clinical testing and as therapeutic biomarkers for numerous diseases. Accumulating clinical evidence indicates that microRNAs are encompassed by extracellular bodies, such as exosomes, that affect the surrounding environment and cells. Furthermore, microRNAs have been shown to retain stability in transformed serum. In view of their simultaneous effects on multiple target genes and strong correlations with clinical parameters and patient survival, microRNAs present a valuable tool for non-invasive detection of diseases. MicroRNAs are currently under investigation as clinical drugs for several diseases, including liver cancer. In this review, we discuss the roles of microRNAs in liver cancer and highlight the importance of specific microRNAs in liver cancer progression. We further review the development of microRNA-based clinical formulations (biomarkers and therapeutic drugs) and compare them with traditional protein-based approaches. We discuss challenges in the development and clinical application of microRNAs and explore why the current miRNA drugs aimed at treating liver cancer have not progressed smoothly in clinical trials. Finally, we offer suggestions and guidelines that could facilitate future progress in this field.

## 2. Pathological Characteristics and Clinical Development of HCC

Hepatocellular carcinoma (HCC) is the second most common malignant cancer type in terms of fatality ranking [1]. Since the 1970s, the incidence of liver cancer has gradually increased, spreading from the Asian countries to several Western countries and the Northern hemisphere. Moreover, HCC is more common in males aged 40–50 years than women [2].

The occurrence of HCC is a continuous and slow process. The majority of cases of HCC are initially caused by hepatitis, non-alcoholic fatty liver disease, and alcohol-related fatty liver disease [3], induced when liver cells are exposed to risk factors that cause long-term damage, including chronic hepatitis B (HBV virus-induced) or hepatitis C (HCV virus-induced), excessive drinking, fatty liver due to various metabolic factors, and toxins produced by food spoilage (such as chrysanthemum toxin or aristolochic acid) [4]. During the process of repeated damage and repair of liver cells, the instability of chromosomes increases, resulting in mutation and loss of control of both oncogenes and tumor suppressor genes. In normal cells, oncogenes and tumor suppressor genes are in a balanced state, each performing its own function to maintain cell growth. Upon loss of this balance, abnormally regulated genes promote aberrant signal transmission in the cell, in turn triggering dysregulation of metabolism, uncontrolled growth, immune imbalance, inflammation, and other malignant tumor-like changes that lead to the development of tumors over time. Various mechanisms, including epigenetics, exosomes, autophagy, metabolic regulation, and immune suppression, are under investigation in association with HCC, resulting in the gradual functional characterization of several uncontrolled genes [5].

In addition to risk factor exposure and metabolic problems, the performance of certain genes/proteins is also considered to be a key factor affecting whether a person is at risk for liver cancer. Understanding the genetic background of HCC will be crucial for the development of new therapies aimed at selected targets. With the help of human genomics technology, researchers have uncovered some gene expression patterns that are characteristic of liver cancer [6,7]. The genes that are altered under liver cancer can be roughly divided into two categories: The first category comprises genes that are altered by or related to risk factors, such as TP53 mutations caused by aflatoxin exposure and KRAS mutations related to vinyl chloride exposure. The second category comprises genes that are involved in etiologically unspecified changes, such as activation of the WNT/CTNNB1 pathway through mutations in CTNNB1 and/or axis inhibition protein (AXIN), and inactivation of the retinoblastoma and insulin-like growth factor 2 receptor (IGF2R) pathways through inactivation of retinoblastoma 1 (RB1) [8]. These novel findings and the readily available genetic resources and analytical tools may be keys to unraveling the molecular basis of HCC. Clinically, multiple strategies have been developed for the different stages of the disease although the staging methods of HCC vary across countries. The commonly used methods are Cancer of the Liver Italian Program (CLIP) score, Barcelona Clinic Liver Cancer (BCLC) staging, Tumor/Node/Metastasis (TNM) classification system, and Hong Kong Liver Cancer (HKLC) staging [9]. According to the management consensus guideline for HCC, different stages of HCC should be addressed by different treatment strategies, such as surgery, local ablation, transarterial therapies (including transarterial chemoembolization (TACE)), systemic treatment, radiotherapy, and prevention [10]. However, we currently lack an effective systemic chemotherapy, immunotherapy, or targeted therapy for patients with advanced HCC. Even the approved targeted drug, sorafenib, does not significantly improve the survival rate of patients [11]. 

Traditional treatment strategies for HCC include surgical resection, liver transplantation, minimally invasive locoregional therapies (percutaneous ablation, transarterial chemoembolization (TACE), and transarterial radioembolization (TARE)), and systemic chemotherapy drugs, such as etoposide, doxorubicin, cisplatin, 5-fluorouracil, and leucovorin [12,13]. The novel concept of targeted drugs has emerged in recent years. The majority of these drugs are used for patients with advanced HCC, such as sorafenib, lenvatinib, regorafenib, nivolumab, cabozantinib, and ramucirumab [9]. However, the current outcomes of HCC treatment remain unsatisfactory. Although the underlying reasons are complex, the poor results may be mainly attributable to consistently late diagnosis due to a lack of good clinical practice and biomarkers that can accurately predict risk of disease in the early stages, missing the optimal treatment period.

## 3. The Biosynthesis and Action Mechanisms of MicroRNAs

**Figure 1:**
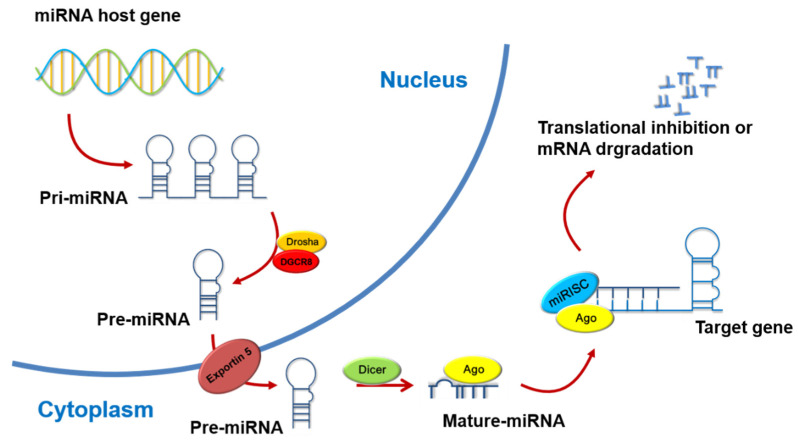
The biosynthesis and action mechanisms of microRNA.

The first miRNA, lin-4, was discovered in *Caenorhabditis elegans* in 1993. Since then, the field of molecular biology has undergone drastic changes. Today, new miRNAs are still being discovered, and the roles of microRNAs in gene regulation are well recognized [14]. MicroRNAs were initially identified during exploration of targets that could be potentially applied as disease biomarkers. MicroRNAs (miRNAs) are 17–22 nt non-coding, single-stranded RNA molecules that play key roles in post-transcriptional gene regulation [15]. Primary miRNAs (pri-miRNAs) are transcribed by RNA polymerase II. From the pri-miRNA, a stereotypical stem-loop hairpin precursor miRNA (pre-miRNA) is cleaved in the nucleus by a complex consisting of the RNA-binding protein, DiGeorge syndrome critical region 8 (DGCR8), and the ribonuclease III enzyme, Drosha [16], which cleaves the pri-miRNA duplex at the base of its characteristic hairpin structure. The generated pre-miRNA is exported to the cytoplasm by the Exportin 5 complex. In the cytoplasm, the RNase III enzyme, Dicer, cleaves the pre-miRNA by removing the terminal loop, thereby generating the mature miRNA, which is then released from pre-miRNA. The biologically active mature miRNA strand is ATP-dependently loaded to a protein of the Argonaute (AGO) family, which, along with the guide strand, forms the miRNA-induced silencing complex (miRISC). The target specificity of miRISC is due to its interaction with complementary sequences in the 3′ untranslated regions (3’UTRs) of the target mRNAs, called miRNA response elements (MREs). The degree of MRE complementarity determines whether there is AGO2-dependent slicing of the target mRNA or miRISC-mediated translational inhibition and target mRNA decay [17]. A fully complementary miRNA–MRE interaction induces AGO2 endonuclease activity and targets mRNA cleavage (Figure 1) [14,18]. Recent research has gradually clarified the relationships between miRNAs and other non-coding RNAs (ncRNAs) [19]. These ncRNAs use the characteristics of their nucleic acid sequences to affect gene expression in cells, from stacking into different three-dimensional structures (scaffold) to adsorbing transcription factors, even affecting chromosomal modification. The mechanism of action of miRNAs involves using the complementary characteristics between nucleic acids, which, in turn, affects their post-transcriptional effects. NcRNAs are also nucleic acid sequences that bind miRNA [20]. Following the discovery of miRNAs in plants, further studies gradually revealed that miRNAs extensively regulate more than 60% gene expression in the human genome and strongly participate in various physiological phenomena essential to maintain life, such as differentiation, proliferation, apoptosis, and development. Notably, dysregulation of miRNAs is significantly associated with various diseases, including muscular dystrophy, diabetes, and several cancer types [21].

The biosynthesis of miRNA begins at nuclear chromosomes, where the primary miRNA (pri-miRNA) is transcribed by RNA polymerase II and then sheared by the nuclear RNase III enzyme, Drosha, and its co-factor to form a precursor miRNA (pre-miRNA). The pre-miRNA is sent out of the nucleus via Exportin 5, and the mature miRNA is formed by Dicer and other cytoplasmic complexes. The mature miRNA combines with Argonaute (AGO) to form an RNA-induced silencing complex (RISC), which interacts with the 3′-untranslated region (3′UTR) of a target mRNA. There, sequence complementarity determines whether the target mRNA will be destabilized or degraded, thereby affecting the performance of genes.

## 4. The Important Roles and Functions of MicroRNAs in HCC

Since miRNAs can affect cell physiology, they play unique roles in cancer cells at different stages. In-depth knowledge of various miRNAs can improve the likelihood of identifying those with clinical diagnostic or therapeutic value [22]. One basic strategy is to characterize specific miRNAs that are dysregulated in tumors. Further studies are required to establish whether miRNAs circulating in human body fluids could serve as key biomarkers [23]. 

MicroRNA-122 (miR-122) is a representative liver-specific miRNA candidate with potential clinical significance. MiR-122 can be used to not only distinguish between healthy individuals and liver cancer patients but also liver cancers induced by different virus types [24], based on its distinct effects on hepatitis C virus (HCV) and hepatitis B virus (HBV). Specifically, miRNA-122 increases the rapidity of HCV replication while suppressing HBV replication [25]. Recent studies have demonstrated that interactions of miR-122 with HCV RNA induce conformational changes in the 5′UTR internal ribosome entry site (IRES) structure to form a stem loop II structure (SLII) and trigger hijacking of the translating 80S ribosome through binding of SLIII to the 40S subunit, leading to efficient translation [26]. At the same time, viral protein R-mediated regulation of HCV replication depends on the host protein DDB1-Cul4 associate factor 1 (DCAF1), supporting the involvement of DCAF1 in the replication of HCV. Measurement of the expression levels of miR-122 and target CAT-1 mRNA revealed that miR-122 was downregulated following DCAF1 repression. Furthermore, overexpression of miR-122 rescued impairment of HCV replication induced by DCAF1 repression, suggesting that DCAF1 is involved in HCV replication through regulation of miR-122 [27]. Several studies suggest that miR-122 is necessary for RNA replication of HCV in the liver [28]. In earlier phase II clinical trials, treatment of HCV patients with oligonucleotides sequestering miR-122 resulted in a significant loss of viral RNA [29]. Therefore, miR-122-related treatment guidelines have been a long-term focus of research into the therapeutic options for HCC patients infected with HCV. In comparison, studies on the role of miR-122 in HBV-infected HCC are relatively rare. However, the data obtained to date consistently indicate that miR-122 is downregulated in patients with HBV-associated HCC and related to tumor size, lymph node metastasis, TNM stage, pathological type, differentiation grade, liver cirrhosis, AFP, and HBV DNA [30,31]. These converse effects further support the potential clinical utility of miR-122 in effectively distinguishing between HBC- and HCV-infected patients with HCC. 

In addition to miR-122, several reports suggested that some miRNAs, such as miR-21, miR-125, and miR-199 family members, are implicated in HCC [30,32]. MiRNA 199a/b-3p, the third most highly expressed miRNA in liver, is consistently downregulated in patients with HCC associated with HBV infection, HCV infection, and alcohol consumption [33]. Furthermore, expression of miR-199 family members is suppressed in HCC tumors and cell lines [34]. Low miR-199a/b-5p expression is also associated with poor overall survival of HCC patients. MiR-199a/b-5p overexpression in HCC cell lines has been shown to inhibit cell proliferation, migration, and invasion, both in vitro and in vivo [35]. A strong correlation exists between miR-199a-5p and miR-199a-3p in HCC specimens and miR-199a-5p additionally contributes to E-cadherin regulation, underlying the complex network of interactions involving miR-199a and its influence on tumor aggressiveness. These findings support the restoration of physiologic levels of miR-199a-3p as a possible therapeutic strategy for HCC [36]. In terms of the molecular mechanism of action, regulators of G-protein signaling (RGS) are critical for defining G-protein-dependent signal fidelity and RGS17 plays an important regulatory role in cancer cell proliferation, migration, and invasion. Overexpression of miR-199 significantly suppresses HCC cell proliferation, migration, and invasion, which may be achieved via inhibition of RGS [37]. 

MiR-21 is one of the most frequently upregulated miRNAs in liver diseases, such as NAFLD and HCC, and associated with poor overall survival. MiR-21 is reported to promote nonalcoholic steatohepatitis-related HCC (NAHCC) and other liver diseases via several mechanisms, such as inducing increases in Phosphatase and Tensin Homolog B (PTENB), Peroxisome Proliferator-Activated Receptor alpha A (PPARAA), and activation of the PI3K/AKT pathway. Additionally, miR-21 is reported to induce hepatic inflammation through promotion of inflammatory gene expression via the STAT3 signaling pathways, leading to liver disease [38]. High miR-21-3p levels are positively associated with advanced tumor stages [39]. MiR-21-3p promotes metastasis of HCC cells and upregulation of Yes-Associated Protein 1 (YAP1) expression via direct inhibition of SMAD7, which represents a major epigenetic mechanism in the pathogenesis of HCC [39]. High-Mobility Group Box 1 (HMGB1) and Cluster Differentiation 44 (CD44) additionally play critical roles in HCC progression. Activation of RAGE/JNK signaling by extracellular HMGB1 underlies overexpression of miR-21 [40]. 

MiR-214 is an interesting miRNA that functions either as an oncogene or tumor suppressor in different cancer types [41]. MiR-214 has been clearly identified as a tumor suppressor in HCC [42] that can prevent tumor development through β-catenin suppression, which presents a novel option for HCC therapy [43]. Additionally, miR-214 suppresses cell proliferation, migration, and metabolism via targeting Pyruvate Dehydrogenase Kinase Isoform 2 (PDK2) and PHD Finger Protein 6 (PHF6) in HCC [41]. In addition to the coding genes, miR-214 affects long non-coding RNAs (lncRNA). In HCC, the long non-coding RNA, PVT1, is linked to malignancies and may serve as a deleterious therapy target. MiR-214 has been identified as a crucial negative regulator of PVT1. High miR-214 levels were found to be significantly correlated with diminished PVT1 expression in HCC specimens and silencing of PVT1 via ectopic miR-214 or siRNA markedly inhibited the viability and invasive ability of HCC cells [44]. 

MiR-29 acts as a tumor suppressor in HCC. Expression of miR-29 is significantly reduced in HCC tissues and cell lines and low miR-29 levels are associated with disease progression and shorter patient survival times [45]. Thus, miR-29 has been highlighted as a potential biomarker for non-invasive diagnosis of NAFLD [46]. In terms of mechanism of action, the miR-29 family functions as tumor suppressors by targeting Ribosomal Protein S15a (RPS15A) and regulating the cell cycle in HCC [47]. Moreover, miR-29b has been identified as a negative regulatory target gene of the lncRNA HLA Complex P5 (HCP5), which plays a tumor suppressor role to prevent proliferation, migration, and invasion of HCC cells [48]. 

Unlike the coding RNAs inhibited by microRNAs, many ncRNAs can interfere with binding of microRNAs to their target genes, thereby producing the opposite effect, based on the mechanism utilized by miRNAs of using the complementary characteristics of the nucleic acid sequences and target genes to affect the stability of their RNA or efficiency of translation. A combination of ncRNA with microRNA reduces the likelihood of a particular microRNA directly acting on its target gene, typically designated a ‘microRNA sponge’ [20].

Colorectal Neoplasia Differentially Expressed (CRNDE) is an upregulated lncRNA in HCC positively correlated with poor clinical outcomes. Suppression of CRNDE has been shown to induce a marked decrease in HCC cell proliferation, migration, and chemoresistance. CRNDE interacts directly with Enhancer of Zeste Homolog (EZH2), Suppressor of Zeste 12 (SUZ12), and Suppressor of Variegation 3-9 Homolog 1 (SUV39H1) and mediates their inhibition of tumor suppressor genes [49]. CRNDE is additionally reported to promote proliferation and metastasis by acting as a miR-539-5p sponge to regulate POU Class 2 Homeobox 1 (POU2F1) expression in HCC [50]. 

Expression of the lncRNA H19 decreases after birth in the majority of tissues, but is re-expressed in many cancer types. Notably, H19 expression is higher in women than men. H19 is positively related to liver cirrhosis and negatively correlated with the survival rate of HCC [51]. Furthermore, H19 has been shown to have an adverse effect on sorafenib therapy for liver cancer, inhibiting the sensitivity of liver cancer cells to sorafenib via upregulation of miR-675 [52]. The miR-200 family plays a key role in regulating the epithelial–mesenchymal transition (EMT) of cells. Moreover, miR-200 is highly related to tumorigenesis and significantly inhibited in HCC [53]. A microRNA sponge relationship between miR-200 and H19 has been reported. H19 promotes HCC bone metastasis by reducing osteoprotegerin (OPG) expression, which is mediated by Protein Phosphatase 1 Catalytic subunit alpha (PPP1CA)-induced inactivation of the p38/MAPK pathway along with sponging miR-200b-3p [54].

**Figure 2:**
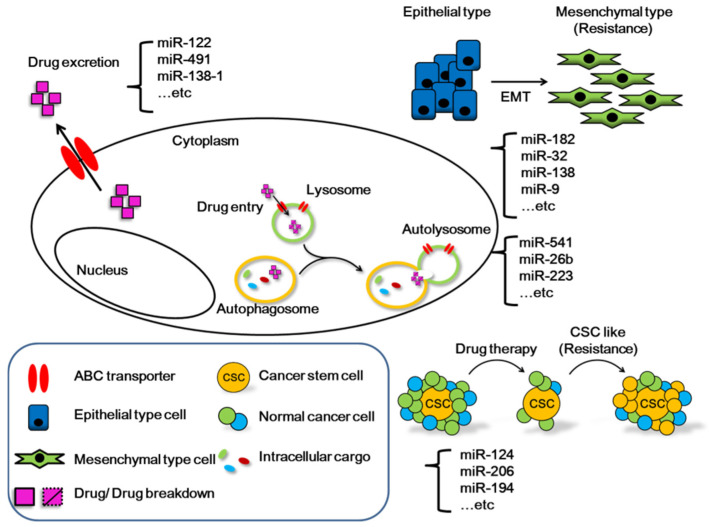
Mechanisms of action of the microRNAs involved in drug resistance of HCC cells.

While specific clinical treatments for HCC are lacking, a number of chemotherapy drugs show significant benefits for a proportion of patients with advanced disease [55]. Genetic heterogeneity plays an important role in the effectiveness of drugs against cancer cells. For this reason, identification of high-quality biomarkers is valuable in the diagnosis of cancer. At the same time, a choice of different drugs is urgently required [56]. Accumulating evidence of the participation of numerous miRNAs in the drug resistance process of cancer cells highlights the importance of a comprehensive understanding of their physiological functions (Figure 2) [57].

In HCC cells, miRNAs are widely involved in various mechanisms related to drug resistance through elimination of drugs from the ABC transporters in the cell, lysosomes and autophagy that degrade drugs within the cell, or EMT and CSC that directly affect cell morphology.

Dysregulation of autophagy is a double-edged blade in cancer. Upon exposure of normal cells to adverse environments and damage, autophagy is activated to reduce stress for avoiding mutations and generating cancer cells. However, under conditions where cancer cells are formed, autophagy can induce EMT, regulate metabolism, and mediate drug resistance. Multiple genes, RNA molecules, proteins, and specific drugs exert antitumor effects by inhibiting autophagy-mediated drug resistance [58]. For instance, miR-541 is downregulated in HCC and associated with malignant clinicopathologic phenotypes (growth, metastasis, and autophagy), recurrence, and survival of patients with HCC [59,60]. Dysregulation of miR-541 through the autophagy-related gene 2A (ATG2A) and Ras-related protein Rab-1B (RAB1B) axis plays a critical role in patient responses to sorafenib treatment. Manipulation of this axis may benefit survival of patients with HCC, especially in the context of highly pursued strategies to eliminate drug resistance [60]. 

Expression of miR-26b-5p is decreased in HCC and associated with poor survival [61]. Zinc ribbon domain-containing 1 (ZNRD1) is frequently upregulated in HCC compared with non-tumor tissues. High ZNRD1 expression in HCC tissues is positively associated with advanced tumor stage and poor prognosis. MiR-26b directly inhibits the transcriptional activity of ZNRD1 and Wnt/β-catenin signaling to suppress HCC development [62]. Moreover, miR-26b has been shown to enhance HCC cell sensitivity to doxorubicin through suppressing Ubiquitin Specific Peptidase 9 X-Linked (USP9X)-mediated p53 de-ubiquitination caused by DNA-damaging drugs and autophagy regulation [63]. Expression of miR-223-3p is markedly lower in cancer tissues relative to their non-cancerous counterparts in HCC. Serum miR-223-3p has been identified as an independent prognostic factor of overall survival in HBV-related HCC [64]. Furthermore, miR-223 overexpression is reported to inhibit doxorubicin-induced autophagy by targeting Forkhead box class O 3a (FOXO3a) and reverse chemoresistance in hepatocellular carcinoma cells [65].

Several factors underlie chemotherapy resistance in cancer. In addition to autophagy, ABC transporters related to membrane transporters can promote drug resistance. In addition, other factors through inducing changes in cell morphology, such as EMT and cancer stem cells, are implicated. Changes in drug resistance triggered by these different mechanisms are significantly related to microRNAs [66]. 

Multi-drug resistance (MDR) is a major obstacle in cancer treatment. The primary mechanism underlying acquired chemoresistance is overexpression of adenosine triphosphate-binding cassette (ABC) transporters. Dysregulation of miRNA is another common factor contributing to this phenotype [67]. For instance, miR-122 is an important microRNA in nature that affects drug resistance. Mechanistically, miR-122 modulates the sensitivity of cells to doxorubicin through downregulation of MDR-related genes, ABCB1 and ABCF2, and inhibits HCC growth by inducing cell cycle arrest [68]. Additionally, miR-122 inhibits MDR1 (ABCB1) expression via suppression of the Wnt/β-catenin pathway, thereby enhancing HCC sensitivity to oxaliplatin (OXA) [69]. Expression of another microRNA, miR-491, in HCC tissues is significantly lower than that in tumor-adjacent tissues and correlated with malignant clinicopathological features [70]. MiR-491 is additionally involved in the ABCB1-mediated doxorubicin and vinblastine resistance of HCC. Specifically, miR-491 downregulates ABCB1 and its transcription factor Sp3 through direct targeting of their 3’UTR regions [71]. p21-Activated Kinase 5 (Pak5) contributes to sorafenib resistance of HCC via regulatory effects on the β-catenin/ABCB1 signaling and miR-138-1-3p is downregulated in sorafenib-resistant HCC cell lines. Earlier studies indicate that miR-138-1-3p reduces protein expression of PAK5 by directly targeting its 3’UTR [72].

Epithelial–mesenchymal transition (EMT) refers to the process of critical morphological transformation of cancer cells during metastasis. EMT is achieved through alternation of epithelial and mesenchymal forms, including changes in apical–basal polarity and intracellular junctions and gain of mobility through increasing or decreasing the number of genes directed by a small number of transcription factors. Accumulating studies suggest that in addition to the risk of metastasis, the type of EMT is related to drug resistance of cancer cells [73]. EMT has additionally been implicated in stem cell properties and therapeutic resistance of cancer cells [74]. The lncRNA POIR was initially identified during a study on sorafenib resistance. Further research disclosed that POIR has a miRNA sponge effect through direct binding to miR-182-5p, leading to promotion of EMT changes and a strong ability to resist sorafenib therapy [75]. MiR-32 is associated with tumor progression and poor prognosis in several diseases, including HCC [76]. This miRNA downregulates phosphatase and tensin homolog (PTEN) through direct targeting of its 3’UTR, affecting proliferation, migration, and invasion of HCC cells via the PTEN/Akt signaling pathway [77]. Using a similar mechanism, miR-32 promotes resistance of HCC cells to 5-Fluorouracil through EMT and angiogenesis [78]. Other miRNAs, such as miR-138 and miR-9, interact with their respective target genes in HCC, specifically, Enhancer Of Zeste 2 Polycomb Repressive Complex 2 Subunit (EZH2) and Eukaryotic Translation Initiation Factor 5A2 (EIF5A2), to affect EMT and induce cisplatin sensitivity [79,80].

Cancer stem cells (CSC) are also considered tumor-initiating cells (TIC). Recent studies suggest that their strong resistance to chemotherapy is related to recurrence of cancer, mainly based on the ability of CSCs to self-renew and differentiate into heterogeneous lineages [81]. MiR-124 acts as a tumor suppressor through regulation of cyclin D1 and cyclin-dependent kinase 6 (CDK6), which may serve as a potential therapeutic target in HCC [82]. This miRNA is downregulated in liver CSC and associated with prognostic survival in HCC. Functionally, miR-124 inhibits the self-renewal ability of liver CSC and reduces tumor occurrence. In particular, miR-124 has been shown to promote response to sorafenib by regulating the performance of the target gene Caveolin-1 (CAV1) [83]. Another miRNA, miR-206, inhibits tumor progression in HCC, reducing proliferation, invasion, and migration and promoting apoptosis of tumor cells through regulatory effects on Protein Tyrosine Phosphatase 1B (PTP1B) and cMET [84,85]. In addition, miR-206 expression is reduced in both chemoresistant and recurrent HCC cases. Mechanistically, miR-206 inhibits liver CSC expansion by suppressing dedifferentiation of HCC cells and attenuating self-renewal of liver CSCs through direct targeting of Epidermal Growth Factor Receptor (EGFR) [86]. Another similar miRNA, miR-194, has a lower performance in cells with higher chemical resistance and affects the self-renewal ability of CSC through targeting Ras-related C3 botulinum toxin substrate 1 (RAC1), consequently affecting sensitivity to sorafenib [87].

## 5. Application of MicroRNAs to Clinical Technology

**Figure 3:**
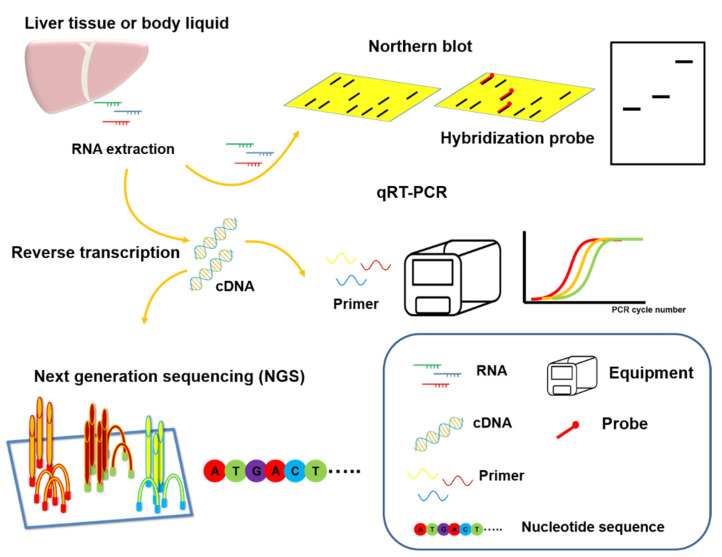
Typical techniques for detecting miRNAs.

The traditional methods of miRNA detection include Northern blotting, microarray, quantitative real-time polymerase chain reaction (qRT-PCR), and next-generation sequencing (NGS). Research in recent years has focused on the use of qRT-PCR and NGS or the development of related or extended detection methods based on these technologies (Figure 3) [88]. The traditional miRNA detection methods have considerable drawbacks. For example, Northern blotting requires the detection of large amounts of RNA and has high technical thresholds. At the same time, it is impossible to detect differences in signals from RNA molecules of the same molecular weight. However, qRT-PCR is limited by the accuracy of the primer design and RNA samples need to undergo complex processing for conversion into reactive cDNA, which greatly increases the chances of error. However, modern NGS technology faces some issues, such as the length of time required, robustness of the database, and influence of the structure and composition (secondary structure or GC content) of RNA [89]. For clinical application of miRNAs, solutions to address these technical deficiencies, such as isothermal amplification, paper-based, oligonucleotide template reaction, nanobead-based, electrochemical signaling-based, and microfluidic chip-based strategies, are under development [90]. Because of the high diagnostic value of miRNAs, to achieve specific applications in practice, the development of highly accurate, rapid, and low-cost detection methods continues to be a focus of research. In addition, miRNAs hold significant promise as blood-borne and circulating biomarkers for numerous diseases. However, the reliability of such liquid biopsies is significantly affected by problems associated with the handling of biological liquids in the pre-analytical stages of biomarker processing [91].

In the clinic, RNA (including miRNA) is isolated and extracted from a patient’s tissue sample or body fluid. In addition to direct analysis of RNA using a hybridizing probe (Northern blot analysis), the RNA may be reverse transcribed to form cDNA. Real-time quantitative reverse-transcription PCR (qRT-PCR) uses transcript-specific primers to amplify the cDNA and assess the presence and level of gene expression. Next generation sequencing (NGS) is a high-throughput technology that can be used to analyze cDNA sequences, compare detected sequences, and obtain data on differences in gene expression.

## 6. The Advantages of MicroRNAs for the Diagnosis and Treatment of HCC

Precise and personalized therapy is a major trend in current and future medicine. Appropriate medical strategies and risk assessments can be formulated based on specific biological parameters and comparative analysis of clinical information. For this reason, biomarkers have become a major focus of research. The ideal characteristics of cancer-related biomarkers are as follows: specific responses to carcinogen-related pathological changes, sufficient expression in the body for easy detection, and adequate specificity to avoid excessive possibility of blurring the focus. In clinical oncology, biomarkers for early diagnosis are critical for not only preventing the incidence of cancer but also establishing effective therapeutic strategies [92]. 

**Figure 4:**
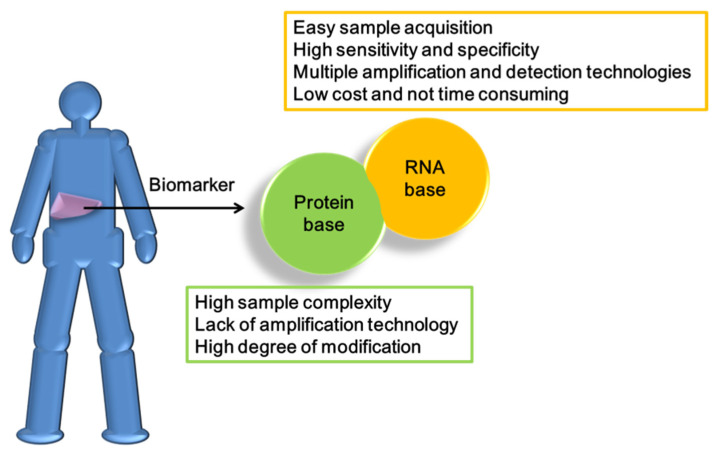
Advantages of RNA as biomarkers in clinical testing.

Early research to develop biomarkers based on proteomics was limited by the evolution of technology, with disappointing advances in the development of diagnostic equipment. Proteosome-related biomarkers have several shortcomings, including limitations in analysis of complex biological materials and inherent statistical challenges in high-dimensionality data sets populated by comparatively few samples. Moreover, selection of protein-type biomarkers for research and development is difficult. The main technical problem clearly lies in the inability to amplify the product for operation. Immunoaffinity capture is the most effective procedure for detection and quantification of protein biomarker candidates present at or below the nanogram/milliliter levels in blood, where many disease-specific biomarkers are located. However, the comprehensiveness of antibody and other protein profiling arrays, even for unmodified proteins, does not currently begin to approach what is available for transcriptional profiling, rendering this approach unsuitable for true de novo discovery efforts (Figure 4) [93]. Protein biomarkers, such as AFP and CEA, are mainly used to detect the risk of HCC and not applied alone for evaluation owing to their lack of specificity [94,95].

The development and use of RNA as HCC biomarkers has significant advantages compared with protein. Regardless of the preparation and analysis of clinical specimens, RNA has higher economic value and can be used in multiple technologies.

RNA is an important component of the central dogma and can transmit genetic information by participating in transcription and post-transcriptional regulation events, reliably reflecting various physiological phenomena. During the search for biomarkers, inclusion of the RNA system for development was naturally considered. However, application of RNA has a number of difficulties, the most typical being serious instability derived from its single-stranded structure. In terms of advantages, RNA can be detected, even at very low levels, and effectively used as a biomarker with high-throughput technology for various diseases. RNA is also widely present in various biological fluids, such as serum, saliva, and urine, and therefore has the advantage of non-invasive detection, which can be applied to monitor tumor progression and response to treatment. Compared with protein biomarkers, RNA biomarkers are more specific and sensitive. RNA can also be amplified with PCR along with other technologies. In terms of cost, each protein biomarker needs to use the corresponding antibody for operation while RNA biomarkers only require the relevant primers and are therefore more affordable. Moreover, compared to DNA biomarkers, RNA biomarkers have the added advantage of providing dynamic insights into the cellular states and regulatory processes [96]. 

Since RNA instability is a significant challenge, miRNAs may enhance the application value of RNA-based markers. Previous studies indicate that miRNAs are remarkably stable in plasma and serum as well as extreme pH and multiple freeze–thaw cycles, as well as being resistant to RNase activity [97]. The cell specificity, richnessm and stability of miRNA molecules enhance their clinical value. Notably, about 10% of circulating miRNAs are secreted in extracellular bodies and other parts bind specific proteins to form complexes, such as Argonaute 2 (Ago2), nucleophosmin 1 (NPM 1), and high-density lipoprotein (HDL). Compared with other RNAs, the stability of miRNAs is therefore greatly increased and these molecules can effectively avoid degradation by RNases. Clarification of the precise origins and functions of various miRNAs may facilitate diagnosis and treatment of different cancer types [98]. MiRNA-guided diagnostics provide a powerful molecular approach for evaluating clinical samples. MiRNA biomarkers offer a useful tool for assessing cancer development owing to their essential roles in nearly all cellular pathways governing human malignancies, such as carcinogenesis, cancer progression, cell invasion and metastasis, cell survival, and response to therapeutic drugs [99].

## 7. The Disadvantages of MicroRNAs in the Diagnosis and Treatment of HCC

In clinical applications, miRNAs circulating in the body display a high degree of specificity and sensitivity as biomarkers and are valuable for diagnosis and monitoring of recovery. However, miRNA-based strategies pose a number of problems that need to be overcome. First, the same specific miRNA biomarkers have been frequently detected in patients diagnosed with different types of cancer. In particular, miRNAs expressed in tumor tissues are more serious than those circulating in the body. Several miRNAs are reported to be abnormally expressed in tumor tissues but cannot effectively distinguish between benign and malignant tissues. For instance, miR-21-5p is strongly correlated with multiple types of cancer but cannot discriminate between malignant tumors and benign polyps in colorectal cancer (CRC). At the same time, miRNAs may have distinct functions and expression patterns in different cancer tissues. In patients with non-small cell lung cancer (NSCLC), HCC, and gastric cancer, miR-21-5p is reported to be upregulated and, conversely, downregulated in patients with breast cancer, signifying different contributory roles in distinct cancer types [100,101]. 

Compared with RNA, miRNAs are more suitable as biomarkers owing to their stability based on associations with extracellular vesicles, which facilitates avoidance of RNase-mediated digestion, along with the convenience of non-invasive testing. However, extraction and analysis of miRNAs from extracellular vesicles present considerable challenges. During the storage period, miRNAs are released from blood cells, which could lead to false results that significantly differ depending on whether plasma or serum is used. Therefore, the protocols should be standardized for sample collection, storage, and processing. EDTA, citrate, and heparin are commonly used anticoagulants for plasma collection. Some anticoagulants, such as heparin, are known to inhibit reverse transcriptase and DNA polymerase activities [101].

MiRNA detection technology has several limitations that need to be addressed. In general, miRNA detection is performed via qRT-PCR, droplet digital PCR (ddPCR), microarray, and miRNA sequencing. For example, qRT-PCR analysis is classified into absolute and relative quantification methods. However, no constant standards are available for equalization calculation in plasma or blood, leading to difficulties when performing quantitative actions. Conventionally, relative expression of miRNAs mainly relies on small nuclear (e.g., U6) or small nucleolar (e.g., SNORD44) RNAs for normalization. However, analysis procedures for numerous well-known miRNAs are unavailable, such as miR-16, miRs-10b, miR-30a, miR-30d, miR-103, miR-148b, miR-191, and miR-192 [101]. 

Using RNA interference technology, expression levels of proteins can be regulated. This system is a breakthrough that has resulted in the identification of excellent protein targets considered “undruggable” in the past due to technological or other reasons. While microRNAs have unique advantages in clinical diagnosis, significant challenges need to be overcome for use in therapeutic drug development. Oligonucleotide therapeutics have since been developed to specifically silence, restore, or modify the expression of disease-causing or disease-associated genes in cancer and genetic disorders [102]. Several difficulties exist with this type of application, such as degradation of nucleases by biological systems, poor permeability to cell membranes, the need to increase the binding force for complementary sequences, optimization of the delivery method to target tissue, and the occurrence of off-target and unwanted toxicities [103]. Over time, a number of solutions have been developed, including the use of different chemical modifications (phosphodiester linkages, ribose backbone, 2′-O-(2-methoxyethyl), 2′-O-methyl, 2′-locked nucleic acid, and 2′-fluoro) to improve stability and different delivery vehicles to improve transportation (liposomes, polymers, and viruses) [104].

## 8. The Current Clinical Application of MicroRNAs in HCC

In view of the participation of miRNAs in various physiological functions, one documented theory is the exploitation of these molecules for disease screening and treatment development [105]. Biomarker selection should primarily be driven by an attempt to resolve specific clinical issues, such as causative relationship with the disease state [106]. In clinical diagnosis of liver cancer patients, the choice of biomarker is dependent on easy detection (non-invasive) and reflection of different pathological phenomena (tumor stage, size, invasion degree, and patient survival rate) and consideration of specificity is also a significant challenge. AFP, currently the most commonly used clinical biomarker in liver cancer, has inadequate specificity. Biomarkers not only have application value in terms of correlation with pathological phenomena but also aid in selecting and monitoring the effects of drugs for different treatment methods. Based on their intrinsic characteristics, a number of miRNAs have been selected for clinical application in HCC therapy.

MiR-122 is one of the most highly selected candidate miRNAs for HCC owing to its liver specificity [107]. Clinically, miR-122 plays an important role in viral liver cancer and is used to select treatment strategies based on HCV or HBV infection [108]. In addition, miR-122 aids in reducing the shortcomings of AFP detection errors caused by insufficient specificity. By combining the detection of different biomarkers at the same time, more accurate analysis of the different stages and types of liver cancer or hepatitis can be achieved. For instance, combination of serum AFP expression with two potential biomarkers, Glypican-3 (GPC3) and miR-122, shows diagnostic potential for HCV-related early-stage HCC [109], while potent biomarker-based panels comprising serological AFP, miR-122, and circulating telomerase reverse-transcriptase (TERT) promoter mutations can be efficiently used for screening HBV-related HCC [109]. In the selection of treatment strategies, the performance of miR-122 is also used to make an informed choice. Sorafenib is the only first-line treatment approved for advanced HCC. Since many patients experience drug resistance, the development of more effective strategies represents an unmet clinical need [110]. To clarify the molecular mechanisms underlying sorafenib resistance in HCC cells, a miRNA microarray was conducted [111], which revealed a significant reduction in liver-specific miR-122 expression in sorafenib-resistant HCC cells. Further reports suggest that activation of Insulin-like growth factor 1 receptor (IGF-1R) via miR-122 downregulation contributes to RAS signaling associated with drug resistance [111]. 

In addition to miR-122, miR-21 and miR-192 have been highlighted as candidate clinical biomarkers. MiR-21, miR-122, and miR-192 are reported to be differentially expressed among subgroups and positively correlated with AFP levels in HBV-related HCC. Together with AFP, the three miRNAs (miR-21, miR-122, miR-192) may serve as effective biomarkers to improve diagnostic therapy for HCC in HBV-positive patients, in particular, HBV-related liver cirrhosis with normal AFP levels or HCC with small tumor sizes [112]. Similar to miR-122, miR-192 is a specific microRNA located in the liver [113]. MiR-192-5p is abundant in liver tissue where it promotes development of the liver and cellular differentiation and coordinates energy metabolism [114]. Recent studies have demonstrated the involvement of miR-192-5p in several human diseases, especially various tumor types, including lung, HCC, and breast cancer. Notably, miR-192-5p is abundant in biofluids, such as serum and urine, and exosomal levels in the circulation can be used in the diagnosis and prognosis of various diseases, such as chronic hepatitis B infection [115]. Other liver-specific microRNAs include miR-130, miR-183, miR-196, miR-209, and miR-96, potential indicators of liver injury (apoptosis, necrosis, and necroptosis), or hepatitis that display variable expression during acute/fulminant or chronic liver failure, liver fibrosis/cirrhosis, and HCC [116].

In recent years, extensive studies have identified small extracellular vesicles (EV, designated ‘exosomes’) as carriers of various molecules, in particular, miRNA. HBV and HCV use exosomes to spread viral RNA complexes to neighboring human liver cells. Due to their activity in transmitting effector molecules and signals between cells, including RNA, proteins, ncRNA, and DNA fragments, exosomes have attracted significant interest for potential application in the clinic [117]. In addition to the advantages of specificity, non-invasive detection, and stability, miRNAs present in exosomes also have research value [118], such as the clinical candidate mentioned earlier, miR-122. Exosomal miR-122 has been shown to play an important role in HCC and alterations in this miRNA are associated with predictive ability in HCC patients with liver cirrhosis treated with transarterial chemoembolization (TACE). Exosomal miRNAs were isolated from serum samples collected before and after TACE in an earlier study. Expression of this miRNA was significantly decreased after TACE and its expression before TACE was markedly correlated with tumor diameter and Child–Pugh score. According to the median relative expression of miR-122 after and before TACE in liver cirrhosis patients, patients with a higher miR-122 ratio had significantly longer disease-specific survival [119]. In addition to miR-122, miRNAs detected in exosomes in serum with clinical significance are miR-148a and miR-1246, which are more abundantly expressed in HCC relative to the liver cirrhosis and normal control groups [120]. MiR-519d is also considered an excellent candidate biomarker for early diagnosis. Initially, in patient groups with liver cirrhosis, miR-939, miR-595, and miR-519d were shown to successfully discriminate between cirrhotic patients with and without HCC. Further analysis of patient serum miRNAs in the exosome disclosed miR-519d, miR-21, miR-221, and miR-1228 and a correlation between circulating and tissue levels of miR-519d, miR-494, and miR-21 in HCC patients. Among these, miR-519d could be used to distinguish cirrhotic patients without HCC and with early-stage HCC [121]. Earlier studies have evaluated 11 well-known reference genes from circulating exosomes across healthy controls, hepatitis B patients, and HCC patients. A combination of miR-221, miR-191, let-7a, miR-181a, and miR-26a represents an optimal gene reference set to normalize the expression of liver-specific miRNAs for comprehensive investigation into the progression of chronic hepatitis B to HCC, which may be valuable for monitoring hepatitis progression and as a biomarker of early-stage HCC [122]. In addition, the miR-125b and miR-638 levels in exosomes are associated with tumor number, encapsulation, and TNM stage, along with reduced time to recurrence and overall survival. These results support the utility of exosomal miR-125b as a promising prognostic marker for HCC [123,124]. 

Both antagonists and mimics have been developed as miRNA-based therapeutic approaches for cancer. MiRNA antagonists are single-stranded oligonucleotides that hybridize to miRNA complementary sequences and disrupt miRNA activity or processing, with a resultant increase in expression of the target genes (tumor suppressors). MiRNA mimics play a converse role by overexpressing miRNA, leading to downregulation of the target genes (oncogenes; Table 1) [103].

MRX34, a liposomal formulation of miR-34a, is potentially the first class of miRNA mimic cancer therapy. MiR-34a is a naturally occurring tumor suppressor lost or expressed at reduced levels in a broad range of tumor types. Retrospective clinical studies have demonstrated a negative correlation of low miR-34 expression with survival in a number of cancer types. In normal tissue, miR-34a is implicated in the downregulation of over 30 unique oncogenes, including, but not limited to, MET, MYC, PDGFR-α, CDK4/6, and BCL2. Genes involved in tumor immune evasion, such as PD-L1 and DGKζ, are additionally regulated by miR-34a. Exogenous introduction of miR-34a mimics in vitro is reported to suppress cell proliferation, migration, and invasion. Synergistic effects have been observed upon combination of miR-34a mimics with anti-cancer therapies. In pre-clinical animal models, miR-34a delivered by a variety of vehicles inhibited primary tumor growth, blocked metastasis, and improved survival. Moreover, orthotopic mouse models of hepatocellular carcinoma (HCC) displayed significant growth inhibition and tumor regression in more than a third of MRX34-treated animals. Unfortunately, the first human clinical trial of miRNA-based therapy was terminated due to unexpectedly severe immune-mediated toxicity, which resulted in the death of four patients from expansion cohorts [125]. 

Miravirsen (SPC3649), a 15-nucleotide nucleic acid (LNA) containing phosphorothioate modifications, is the first anti-miRNA antisense oligonucleotide (ASO) entered for clinical trials. The modified ASO for treatment of HCV infection targets miR-122, a highly abundant miRNA expressed in the liver that regulates HCV replication [126]. In phase 1 clinical trials, high doses of Miravirsen monotherapy resulted in undetectable HCV RNA levels in some cases. Since Miravirsen is a modified RNA, it naturally accumulates in the liver and does not require a special delivery strategy. Miravirsen is currently undergoing multiple phase 2 clinical trials [127].

Another product developed to target miR-122 in HCV-infected hepatocytes is RG-101, an N-acetyl-D-galactosamine-conjugated RNA antagomiR. Similar to Miravirsen, RG-101 shows considerable efficacy in patients displaying undetectable HCV RNA levels. However, major adverse events, such as severe jaundice, were reported in a recent clinical trial and the FDA has recommended putting the study on hold until clarification of the situation [127].

MiR-103 and miR-107 levels are upregulated in the liver of obese animals and serum of human NAFLD patients. In animal models, miR-107 and miR-103 are reported to regulate insulin sensitivity through direct interactions with caveolin-1 [128]. Recently, RG-125 (AZD4076), a microRNA-103/107 antagonist, entered a phase 1 clinical trial for non-alcoholic steatohepatitis (NASH) treatment [129]. However, this drug has since been associated with a jaundice-related side-effect (known as hyperbilirubinemia) and its development program faces termination.

**Table 1:** MiRNA-based clinical drugs for HCC.

Name	microRNA/Role	Phase Status	Diseases	Therapeutic Agent	Regulation Gene	Side Effect	Reference
MRX34	miR-34/Suppressor	Phase1 (terminated)	HCC	miR-34 mimic	SMAD4, SATB2, PDGFR, c-MET, Axl	Immune-mediated toxicity	[130,131,132,133,134,135]
Miravirsen (SPC3649)	miR-122/Oncogene	Phase2 (suspended)	HCV	Anti-miR-122	Ago2, DCAF1, CAT-1, NIK, LPL, NS5B	No clear side effects	[27,136,137,138,139,140,141]
RG-101	miR-122/Oncogene	Phase2 (discontinued)	HCV	Anti-miR-122		Jaundice	[142,143]
RG-125 (AZD4076)	miR-103/Oncogene miR-107/Oncogene	Phase1	NASH	Anti-miR-103/ 107	HMGB1, P120, ZO-1, LATS2, RGS4, HMGCS2	Hyperbilirubinemia	[144,145,146,147,148,149,150]

## 9. Challenges and Solutions for the Clinical Application of microRNAs

Even though miRNAs have many potential benefits for clinical applications in theory, there are still many challenges to be solved before they can be applied in practice. For example, although the detection of circulating miRNAs in patients’ body fluids would embody a non-invasive mode of analysis, researchers must first address the problem of miRNAs being affected by other substances in the specimen. Since miRNAs circulating in body fluids may be coated by microvesicles, targeted purification methods would also be needed [151]. In addition, many miRNAs become unstable and degraded in the environment within 24 to 72 h in 4 °C or −20 °C, leading to inaccurate detection results [152]. Deviations between tests need to be carefully evaluated.

The total amount of miRNA circulating in body fluids is rarely (if ever) high, which limits the applicability of the analytical methods traditionally used to detect miRNAs, such as qRT-PCR, microarray analysis, and NGS. Although qRT-PCR can amplify the signal of a target sequence, its performance is limited by the basic content of the input. Microarray analysis is not suitable for identifying miRNAs with short sequences and high similarity. High-throughput NGS seems to be the best solution. Although the price is higher, it is suitable for NGS to consider [98,153].

Regarding the use of miRNAs for targeted therapy, the main approach applied to date has been to use an anti-miRNA to silence a target miRNA. However, this strategy has encountered issues related to off-target effects and delivery issues. Among the more than 2000 known miRNAs, only about 200 are considered to have sufficient content and physiological significance to be potentially useful in clinical diagnosis and/or treatment. Moreover, an miRNA comprises only ~20 nucleic acids, of which only ~7 nucleic acids (the so-called seed sequence) can meaningfully combine with a target mRNA. Therefore, many miRNAs have the same target. For example, members of the miR-17 and let-7 families of miRNAs have the same seed sequence. Therefore, even if scientists manage to increase the binding force of an anti-miRNA to a target miRNA, to reduce the influence of other miRNAs by anti-miRNA will be a fundamental problem [154]. 

Some modifications have been proposed to reduce the chance of an anti-miRNA binding to a non-target miRNA. For example, the 2’-OMe-modified miR-93 inhibitor exhibits a reduced chance of mis-binding to miR-106b [155]. Moreover, 2′-MOE-modified LNA, unlocked nucleic acid, and glycol nucleic acid have been shown to efficiently suppress the immune stimulation by anti-miRNA and enhance specificity to reduce off-target induced toxicity [156]. The miRNA that has received the most attention in liver cancer, miR-122, has also been addressed in modification studies [157,158].

Regarding delivery-related issues, many miRNAs have different functions in different tissues, meaning that it would be important to deliver miRNA-based drugs to the right location(s). Although abnormally expressed miRNAs may cause tumors, the same miRNAs can have necessary functions in normal tissues. For example, abnormally high performance of the miR-17–92 cluster can cause lung cancer; however, the lack of this cluster under normal circumstances can cause immune cell dysfunction and developmental disorders [159,160,161]. The current mainstream approach for targeted delivery is to pair a miRNA with a ligand, peptide, or antibody that reacts with a specific antigen on the surface of the target tumor or tissue cells. These targeted delivery approaches can minimize potentially deleterious effects in normal tissues [162,163,164]. Delivery system design must also consider the affinity of the target tissue for the drug. Fortunately, liver tissue is generally amenable to the direct delivery of drugs designed using the basic antisense oligonucleotide (ASO) method, without the need for special carriers [165].

## 10. Discussion

HCC is the most common primary cancer of the liver with extremely high fatality rates. From a clinical viewpoint, the main underlying factor is the lack of an effective and easy-to-implement biomarker for early clinical diagnosis of HCC, which often leads to inadequate treatment outcomes. The suitability of biomarkers is dependent on their accuracy and specificity. Suitable biomarkers should reduce the difficulty of operation and increase patient compliance in addition to being economical. The mainstream biomarker for HCC is AFP. However, a major problem with using AFP for detection is that this protein does not actually reflect the occurrence of HCC, suggesting that AFP is not adequately specific for a diagnosis. A number of previous studies demonstrated no elevation of AFP levels in patients with HCC. Conversely, patients who were not diagnosed with HCC but displayed cirrhosis, cholangiocarcinoma, or other tumors were shown to contain elevated AFP [166,167]. 

In the search for optimal biomarkers, the concept of RNA-based strategies has gradually emerged due to the evolution of biotechnology. Compared with traditional protein biomarkers, the advantages of this method are clear. Clinical samples are difficult to obtain and patient samples should therefore be used cautiously for analysis. However, protein-based biomarkers cannot be employed due to technical limitations. In view of detection sensitivity and amplification properties from a biological viewpoint, the physiological significance of RNA is higher than that of protein. However, the instability of RNA is yet another problem that hinders its application. Based on the theory that non-transcribed RNAs play important physiological roles, the clinical value of miRNAs with a fragment size of only 17–22 nucleic acids has gradually been revealed. Compared with traditional RNA, miRNAs have higher stability and a key ability to simultaneously regulate different genes. At the same time, miRNAs are secreted into human body fluids in different ways, supporting their high potential in non-invasive detection. Nevertheless, a number of problems need to be overcome for optimization of miRNAs as biomarkers for HCC and other diseases in the future. For example, miRNAs have a high degree of tissue specificity. Furthermore, the same miRNAs may play distinct roles in multiple tissue types. The main advantages of miRNA technology include the development of more effective and faster detection methods. However, for methods such as qRT-PCR, several problems exist, such as the need to undergo reverse transcription or inadequate fragment length for effectively identifying differences between similar types of microRNAs. 

In addition to traditional treatments, such as chemotherapy and surgery, the therapeutic options that have received attention in recent years are targeted drugs, such as sorafenib, lenvatinib, and regorafenib [168]. However, HCC is a remarkably heterogeneous disease [169]. Tumor heterogeneity in HCC, such as that found in second primary tumors after curative treatment, synchronous multifocal tumors of different clonality, or intratumor heterogeneity, poses severe challenges for the development and administration of systemic molecular targeted therapies [170]. Considering the problems related to poor efficacy and heterogeneous individual responses to targeted therapy, miRNA expression profiles have been linked to the development of anticancer drug resistance. Therefore, the therapeutic potential of miRNAs may be successfully exploited for overcoming drug resistance in various cancer types. MiRNAs are additionally considered a suitable tool to resolve the issue of resistance to chemotherapy [171]. Research to date suggests that restoring the balance of specific miRNAs in drug-resistant cancer cells should aid in re-establishing sensitivity to drugs. These findings support the potential of miRNAs as co-adjuvants in anticancer therapy. Successive studies have also highlighted abnormalities in numerous microRNAs in patients resistant to sorafenib, such as miR-486, miR-21, and miR-122, which are currently under evaluation [110,172,173]. The clinical potential of miRNAs is undeniable and their paramount roles in drug resistance could be exploited as alternative strategies to manage treatment responses in addition to their utility as predictive circulating biomarkers [174].

Nonetheless, challenges remain in efforts to use miRNAs as a treatment strategy. The most fundamental issue is too many targets for a miRNA effect [175], which is also relevant to the following two failed miRNA drugs. MRX34 is a miR-34a mimic drug for which phase I clinical trials were terminated due to the occurrence of five serious immune-related adverse events [175]. KEGG Orthology-Based Annotation System (KOBAS) analysis revealed that the numerous target genes of miR-34a include many immune pathway-related genes, such as those encoding cytokines and interleukins [175]. This surprising observation likely explains why MRX34 induces such serious immune-related side effects. Another example is the anti-miR-122 drug, RG-101, which progressed to a phase II trial before being discontinued because it induced hyperbilirubinemia in some subjects [175]. As miR-122 is uniquely expressed in the liver, it would seem to be a potentially valid choice for development of a drug that could have few side effects. The target genes of miR-122 include only few genes that are predicted to be related to hyperbilirubinemia. Although this correlation is not sufficient to explain the small amount of hyperbilirubinemia induced by RG-101, it could explain why only a few subjects had side effects until phase II. This highlights the potential impact of the off-target problem. In fact, miRNAs can even act on genes that have incomplete complementation to their seed sequence. Therefore, when selecting miRNAs for drug design, researchers should comprehensively evaluate whether actions on other target genes will induce undesirable side effects. Future efforts could also involve the use of chemical modification and nano packaging materials to increase the binding and accumulation of miRNA drugs within specific tissues [175,176,177].

Although the design of clinical liver cancer drugs based on microRNAs is an interesting concept, numerous issues need to be resolved before clinical realization. In combination with existing target drugs, miRNA technology may provide superior results in the future.

## 11. Conclusions

MiRNAs have an extremely high potential value in clinical applications. In the biomarker context, circulating miRNAs are often coated with extracellular vesicles and exhibit high stability, and thus can be targeted for non-invasive detection. MiRNAs also participate in maintaining the physiological characteristics of various cancer cells, and their levels can reflect the clinical information of many patients. In practical terms, miRNA-based strategies offer technical and cost advantages over traditional protein-based detection technologies. While miRNAs have high clinical value, a number of factors need to be considered to ensure their effective usage as biomarkers, including improving the separation and sampling of extracellular capsules, detection technology, consideration of the appropriate quantitative reference genes, selection of miRNAs that can be clearly detected in body fluids as targets, and development of oncogenic miRNAs that are upregulated in cancer. Ultimately, a panel of selected miRNAs may be more effective in the identification of biomarkers specific for a cancer type. At the same time, further exploration of the basic functions of various miRNAs should be continued to ensure breakthrough prospects [101]. 

Although there has not yet been a successful case of miRNA-based liver cancer treatment, this is still an area of vigorous development. Future research efforts should involve identifying additional miRNAs that are specifically expressed in the liver, to reduce the chance of off-target side effects, as well as the use of chemical modifications, carriers, and/or nanomaterials to improve drug delivery. Moreover, researchers should return to basic research on miRNAs, seeking to understand the many miRNAs whose functions and target genes are not yet known, with the goal of finding more suitable targets for development. Novel miRNA biomarkers could also be used to implement the vision of personalized medicine and provide suitable treatment strategies, thus reducing the unnecessary pain and suffering of patients and achieving more efficient diagnoses and management. The HCC Institute should focus on these guidelines in the future [168].
